# PTSD of Chinese nurses in the normalisation of COVID-19 pandemic prevention and control: Prevalence and correlates

**DOI:** 10.7189/jogh.13.06033

**Published:** 2023-08-25

**Authors:** Xiaofei Mao, Pengfei Luo, Fengzhan Li, Fan Zhang, Jianguo Zhang, Wenxi Deng, Ziqiang Li, Tianya Hou, Wei Dong

**Affiliations:** 1 Faculty of Psychology, Naval Medical University, Shanghai, China; 2 Department of Burn and Trauma Surgery, Changhai Hospital, Naval Medical University, Shanghai, China; 3 Department of Military Medical Psychology, Air Force Medical University, Xi'an, China

## Abstract

**Background:**

Though the severe prevention and control measures faced by Chinese nurses had changed during the normalisation stage of the coronavirus 2019 (COVID-19) pandemic, they still worked under great stress. Due to a lack of related evidence, we aimed to investigate the prevalence and correlates of post-traumatic stress disorder (PTSD) among Chinese nurses during the normalisation of COVID-19 pandemic prevention and control measures.

**Methods:**

Using convenience sampling, we recruited 784 nurses in Jiangsu province, China to complete a survey via their mobile devices. We used a demographic questionnaire, the Perceived Social Support Scale, the Connor-Davidson Resilience Scale, the General Self-Efficacy Scale, and The Impact of Event Scale-Revised to collect data and applied binary logistic regression analysis to identify factors associated with PTSD.

**Results:**

The prevalence of PTSD was 26.4%. Married nurses were less likely to experience PTSD than unmarried ones (odds ratio (OR) = 0.573; 95% confidence interval (CI) = 0.33-0.99, *P* = 0.046). Social support (OR = 0.96; 95% CI = 0.94-0.98, *P* = 0.000) and resilience (OR = 0.98; 95% CI = 0.97-0.99, *P* = 0.004) were significant predictors of PTSD.

**Conclusions:**

PTSD remained prevalent among Chinese nurses as COVID-19 pandemic prevention and control measures became normalised, with an incidence rate of 26.4%. Resilience, social support, and marital status were factors associated with PTSD. Chinese hospital management must intervene to improve resilience and social support for nurses to reduce symptoms of PTSD.

The coronavirus disease 2019 (COVID-19) has led to significant psychological problems for health care staff, especially nurses, who experienced a high mental health burden and had more severe degrees of mental health symptoms during the COVID-19 outbreak [1-4]. Lai et al. [5] found that Chinese nurses had a significantly higher level of depression than physicians during COVID-19 due to a higher risk of infection from their frequent close contact with COVID-19 patients and longer working hours than usual. Moreover, studies have found that the nursing staff experienced more trauma-related stress than other health care workers during the COVID-19 pandemic [6-9]. Specifically, 36.2% of the Chinese nurses were reported to suffer from post-traumatic stress disorder (PTSD) during the COVID-19 pandemic [5], while 36.7% of the Korean nursing participants had a high risk of developing PTSD [10]. Moreover, nursing staff from Italy and South Africa had prevalence rates of PTSD of 39.88% and 40.0%, respectively [11,12].

China has entered the stage of normalised pandemic prevention and control measures in May 2020 [13]. Correspondingly, the national pandemic prevention and control policy shifted from the initial blockade policy into normalised COVID-19 pandemic prevention and control measures [14], while some restrictions, such as regular nucleic acid testing, were still being applied. However, the contents of nurses’ jobs had changed; alongside routine work, they have had to take on regular COVID-19-related responsibilities, including strict hospital admission procedures and treatment of patients from high- and medium-risk areas under the “semiclosed” management mode [15]. Meanwhile, constantly working in stressful environments lead to fatigue among nurses [16]. All these requirements imposed a huge burden and significant workload on nurses, so despite the change in the circumstances which the Chinese nurses faced, they could still be working under significant pressured during the normalised COVID-19 pandemic prevention and control measures.

Considering the lack of studies on the prevalence and correlates of PTSD among Chinese nurses during the normalisation stage, we hypothesised (based on prior studies) that PTSD was highly prevalent in this population and aimed to conduct a cross-sectional study to address this gap.

## METHODS

### Participants and procedures

We conducted a cross-sectional study in January 2022. We included nurses without dyslexia, aged ≥18 years old, and working in a hospital under the normalisation of COVID-19 pandemic prevention and control measures. We excluded those with a history of mental illnesses [17]. Using convenience sampling, we recruited 784 nurses in Jiangsu Province of China after obtaining ethical approval from the Naval Medical University. In their informed written consent document, the participants were assured their responses were anonymous and confidential and were free to withdraw at any time without penalty.

### Measures

#### Demographics

We collected the following demographic information: gender, age, years of working, medical isolation, number of night shifts during the last month, a vaccine against COVID-19, marital status, professional title, employment type, child status, weekly hours of working, working department, and chronic disease condition.

We divided the respondents into two groups (young group: ≤30 years old; middle-aged group: >30 years old) according to their age. We categorised them into three groups by working experience (≤5, 6-10, >10) and two groups by the number of night shifts (<4, ≥4) and working hours (≤40, >40). We grouped medical isolation (yes/no), vaccination status for COVID-19 (yes/no), marital status (married/unmarried (single, divorced, and widowed)), professional title (junior title/intermediate title or above), employment type (permanent contract/fixed-term contract), child status (no child/at least one child), working department (high-risk unit/low-risk unit, with nurses working in fever clinics, COVID-19 medical units, emergency departments as high-risk and the others as low-risk nurses [18]), and chronic disease (yes/no) as binary variables.

#### Perceived Social Support Scale

The Chinese version of the Perceived Social Support Scale (PSSS) is a 12-item self-reported measure assessing the perception of social support [19]. Each item was scored on a seven-point Likert scale, ranging from one (strongly disagree) to seven (strongly agree). We obtained the total score by summing the scores of all items; the score ranged from 12 to 84, with higher scores denoting higher levels of perceived social support. We set Cronbach’s alpha at 0.968.

#### Connor-Davidson Resilience Scale

The Chinese version of Connor-Davidson Resilience Scale (CD-RISC) is a 25-item generic resilience instrument with each item scored on a five-point Likert scale ranging from zero (never) to four (very often) [20]. The total score is calculated as the sum of all questions and ranges from zero to 100, with higher scores indicating higher levels of resilience. The scale has demonstrated good internal and external validity and is widely used by Chinese health care workers [21]. In this study, the Cronbach’s α coefficient of the scale was 0.978.

#### General Self-efficacy Scale (GSES)

The Chinese version of 10-item GSES was used to assess self-efficacy [22]. Items were rated on a four-point Likert scale ranging from one (not true at all) to four (exactly true), with a total score ranging from 10 to 40. Higher scores indicated higher levels of self-efficacy. The scale has been found to have good reliability and validity among Chinese health care workers [23]. In this study, the Cronbach’s alpha coefficient for GSES was 0.917.

#### The Impact of Event Scale-Revised

We used the Impact of Event Scale-Revised (IES-R) to assess post-traumatic stress symptoms caused by traumatic events of COVID-19. The IES-R scale included 22 items and consisted of three subscales: intrusiveness, avoidance and hyperarousal. The total score of the scale ranged from zero to 88. The cut-off scores of nine, 26 and 44 were classified as mild, moderate, and severe distress [24,25]. Respondents with moderate or severe distress were suspected of having PTSD. In the present study, the Cronbach’s alpha was 0.976.

### Statistical analysis

We used the G*Power software, version 3.1.9.7. (Heinrich Heine University Düsseldorf, Düsseldorf, Germany) to estimate the required sample size for this study. We used binary logistic regression analysis to analyse the associations between PTSD and associated factors and employed the F-test (linear multiple regression: Fixed model, *R^2^* increase). We set the effect size (*f^2^*) at 0.15 and the alpha value at 0.05. Approximately 189 participants would provide 95.07% power to detect statistical significance.

We conducted all analyses using SPSS, version 24.0 (IBM, New York, USA), with the significance level set at α = 0.05, and all tests being two-tailed. We applied the Kolmogorov-Smirnov test to check whether the data of anxiety and depression conform to normal distribution. The results showed that the total scores of social support, resilience, self-efficacy, and PTSD were not normally distributed (*P* < 0.001), so we presented the data as medians with interquartile ranges (IQRs). We presented the ranked data which derived from the counts of each level of PTSD as numbers and percentages. We conducted binary logistic regression analysis to detect factors associated with PTSD.

## RESULTS

### Demographic characteristics

We recruited 784 nurses with an average age of 30.38 years (standard deviation (SD) = 6.60). Regarding demographic characteristics, most participants were female (n = 740 (94.4%)), aged 30 or below (n = 490 (63.3%)), had no medical isolation experience (n = 697 (88.9%)), had four night shifts or above during last month (n = 449 (57.3%)), had been vaccinated with COVID-19 vaccine (n = 753 (96.0%)), were married (n = 496 (63.3%)), had junior professional titles (n = 510 (65.1%)), were fixed-term employees (n = 688 (87.8%)), had at least one child (n = 430 (54.8%)), worked in low-risk units (n = 680 (86.7%)), worked more than 40 hours per week (n = 630 (80.4%)), and had no chronic disease (n = 694 (88.5%)) (**Table 1**).

**Table 1:** Demographic characteristics and distribution of PTSD of nurses

Variables	n (%)	Number of subjects with PTSD (n = 207)
Gender		
*Male*	44 (5.6)	13
*Female*	740 (94.4)	194
Age		
*Young group*	496 (63.3)	124
*Middle-aged group*	288 (36.7)	83
Medical isolation		
*Yes*	87 (11.1)	26
*No*	697 (88.9)	181
Night shifts during last month		
*<4*	335 (42.7)	79
*≥4*	449 (57.3)	128
Vaccine shots		
*Yes*	753 (96.0)	197
*No*	31 (4.0)	10
Years of working		
*1-5 y*	295 (37.6)	79
*6-10 y*	254 (32.4)	61
*>10 y*	235 (30.0)	67
Marital status		
*Unmarried*	288 (36.7)	69
*Married*	496 (63.3)	138
Professional title		
*Junior*	510 (65.1)	129
*Intermediate and senior*	274 (34.9)	78
Employment type		
*Permanent*	96 (12.2)	32
*Fixed-term*	688 (87.8)	175
Child status		
*No child*	354 (45.2)	90
*Have children*	430 (54.8)	117
Working department		
*High-risk units*	104 (13.3)	35
*Low-risk units*	680 (86.7)	172
Weekly hours of working		
*≤40 h*	154 (19.6)	35
*>40 h*	630 (80.4)	172
Chronic disease		
*Yes*	90 (11.5)	28
*No*	694 (88.5)	179
Social support, median (IQR)	63.0 (50.0-72.0)	
Resilience, median (IQR)	63.0 (50.0-75.0)	
Self-efficacy, median (IQR)	29.0 (26.0-30.0)	

IQR – interquartile range, PTSD – post-traumatic stress disorder

The median scores for the three study scales were 63.0 (IQR = 50.0-72.0) for the PSSS for social support, 29.0 (IQR = 26.0-30.0) on the CD-RISC for resilience, and 15.0 (IQR = 2.0-26.0) for the GSES for self-efficacy.

### Prevalence and correlates of PTSD

A total of 320 (40.8%), 257 (32.8%), 132 (16.8%), and 75 (9.6%) nurses had normal, mild, moderate, and severe distress, respectively. Therefore, 207 (26.4%) of our participants were suspected of having PTSD.

The results of binary logistic regression analysis showed that nurses who were married were less likely to have PTSD (odds ratio (OR) = 0.57; 95% confidence interval (CI)  = 0.33-0.99, *P* = 0.046)) (**Table 2**). Social support (OR = 0.96; 95% CI = 0.94-0.98, *P* = 0.000) and resilience (OR = 0.98, 95% CI = 0.97-0.99, *P* = 0.004) were significant predictors of PTSD.

**Table 2:** Correlates of PTSD identified by binary logistic regression analysis

Variables	OR	95% CI	*P*-value
Gender			
*Male*	1 (Reference)		
*Female*	1.14	0.52-2.47	0.744
Age			
*Young group*	1 (Reference)		
*Middle-aged group*	0.67	0.303-1.464	0.312
Medical isolation			
*Yes*	1 (Reference)		
*No*	1.26	0.73-2.15	0.406
Night shifts during last month			
*<4*	1 (Reference)		
*≥4*	0.93	0.64-1.34	0.679
Vaccine shots			
*Yes*	1 (Reference)		
*No*	0.61	0.26-1.40	0.243
Years of working			
*1-5 y*	1 (Reference)		
*6-10 y*	2.08	0.83-5.21	0.117
*>10 y*	1.39	0.67-2.92	0.377
Marital status			
*Unmarried*	1 (Reference)		
*Married*	0.57	0.33-0.99	0.046
Professional title			
*Junior*	1 (Reference)		
*Intermediate and senior*	0.74	0.40-1.36	0.328
Employment type			
*Permanent*	1 (Reference)		
*Fixed-term*	1.23	0.69-2.18	0.483
Child status			
*No child*	1(Reference)		
*Have children*	1.28	0.70-2.35	0.425
Working department			
*High-risk units*	1 (Reference)		
*Low-risk units*	1.53	0.926-2.53	0.097
Weekly hours of working			
*≤40 h*	1 (Reference)		
*>40 h*	0.63	0.39-1.00	0.052
Chronic disease			
*Yes*	1 (Reference)		
*No*	1.55	0.93-2.54	0.094
Social support	0.96	0.94-0.98	0.000
Resilience	0.98	0.97-0.99	0.004
Self-efficacy	1.00	0.95-1.05	0.985

OR – odds ratio, CI – confidence interval, PTSD – post-traumatic stress disorder

## DISCUSSION

To the best of our knowledge, this is the first study in China to investigate the prevalence and correlates of PTSD among nurses in the normalisation of COVID-19 pandemic prevention and control measures. In our study, 207 respondents showed moderate and severe distress on the IES-R scale, indicating 26.4% of the nurses might suffer from PTSD. Moreover, marital status, social support, and resilience were significant predictors of PTSD.

Lai et al. [5] investigated PTSD among 764 nurses in 34 hospitals in China from 29 January 2020 to 3 February 2020, and found 36.2% of the nurses suffered from symptoms of PTSD. Recently, a meta-analysis of global prevalence of PTSD during the COVID-19 pandemic reported that 28.22% of the nursing staff had PTSD [8]. Besides, another study from the USA found 29.1% of the nurses had symptoms of PTSD [26]. Notably, the incidence rate of PTSD in our study was lower than that reported in the studies during the outbreak of the COVID-19 pandemic. However, the lifetime, 12-month, and one-month incidence rates of PTSD were 0.30%, 0.20%, and 0.195% in the Chinese general population, respectively [27], which is lower than that in the normalisation of COVID-19 pandemic prevention and control measures.

Though the severe situation faced by nurses has changed in the normalisation phase of COVID-19 pandemic prevention and control, they still worked under high pressure. For example, Zhang et al. [28] found that 38.5% of nurses scored ≥26 on the Perceived Stress Scale, indicating a significant higher level of psychological stress in the post pandemic period in Guangdong Province, China. Besides, 16.1% of the nurses reported moderate to extreme levels of stress under normalised COVID-19 pandemic prevention and control in China [29]. The incidence rate of PTSD in our study indicated that special attention should be paid to Chinese nurses under the normalisation of COVID-19 pandemic prevention and control measures.

Moreover, the results of binary logistic regression analysis revealed that marital status, social support, and resilience were associated with PTSD among Chinese nurses in the normalisation of COVID-19 pandemic prevention and control measures, which was consistent with previous literature [30,31]. Specifically, those who were married, reported higher levels of social support and resilience may experience less symptoms of PTSD in the current study.

Resilience is a process of adapting quickly when facing adversity, trauma, and tragedy, and constitutes a mentality to amend and move forward [32-34]. CD-RISC contains different aspects of resilience, including optimism, sense of control, perseverance to overcome obstacles, sense of humor, and the ability to cope with misfortune and stress, among others. These components of resilience may have protective effects when facing with traumatic events [35-37]. According to previous studies, resilience was negatively associated with PTSD among the Chinese nurses exposed to COVID-19 [31], meaning nurses with higher levels of resilience were more likely to to adjust to the adversity of working with patients infected with COVID-19. Besides, resilience could protect nurses from the risk of developing trauma-related symptoms [38]. Moreover, resilience could be prompted through various programs and training, which have a positive impact on mental health [39]. Therefore, helping nurses cultivate resilience could help reduce the symptoms of PTSD [40].

Social support is defined as “the feeling that one is cared for and has assistance available from other people” and “that one is part of a supportive social network” [41]. It is a protective factor against psychological difficulties. The stress-buffering hypothesis supposes that social support played a role in buffering stress via increasing the levels of self-efficacy and self-esteem [42]. Perceived social support was positively correlated with mental well-being and could help cultivate the ability of overcoming stressful events [43,44], while lacking social support was demonstrated to be a predictor of PTSD in health care workers dealing with the COVID-19 pandemic in a system review [45]. A recent study showed that Korean nurses were more likely to have PTSD if they received low support from managers of nurses during the COVID-19 pandemic [46]. Meanwhile, having high social support was associated with a decline in PTSD among nurses during the COVID-19 pandemic [31] and was a vital protective factor for psychological resilience that maintained mental health and eliminated psychological barriers [47]. Therefore, social support plays an important role in reducing psychological stress, which may protect nurses from traumatic events.

Moreover, we also found married nursing staff were less likely to suffer from PTSD. Prior studies have demonstrated that single Italian nurses reported a higher level of post-traumatic stress than married ones [48,49], possibly due to unmarried ones receiving less support from family [50], suggesting the predictable effect of marital status supported the role of social support to PTSD among nurses indirectly. In such cases, we could conclude that nurses who receive sufficient support from family members would be less likely to have PTSD.

### Implications and limitations

Our study could have relevant implications for identifying and treating PTSD among Chinese nurses in the normalisation of COVID-19 pandemic prevention and control measures, both theoretically and practically. First, this study provided empirical evidence regarding the prevalence of PTSD among the nursing population under the regular COVID-19 pandemic prevention and control measures, suggesting that hospital management should conduct interventions to prevent PTSD in nurses even under the normalization stage. Second, we identified factors associated with PTSD in nurses, which might provide a theoretical basis concerning the influential factors of PTSD under the normalisation stage and point out potential preventive measures. Interventions focusing on promoting resilience and social support must be taken to ameliorate symptoms of PTSD among nurses. Moreover, more care from their colleagues, superiors, and family members is needed for unmarried nursing staff.

Our study also has several limitations. First, we adopted a cross-sectional design, making it difficult to draw causal relationships between PTSD, resilience, social support, and marital status. Longitudinal studies are needed to determine the causality between variables. Second, all participants were from the Jiangsu Province of China. We applied convenience sampling with online distribution of the survey, which may have resulted in a non-representative sample, as filling out the survey took time and effort. Future studies could recruit participants through random cluster sampling with offline distribution from other areas of China to increase the external validity of our findings. Meanwhile, the exclusion criterion regarding the history of mental illnesses in the current study was debatable, which may limit the sample representativeness. Future researchers should focus on psychoses and borderline disorders and only exclude patients with these disorders. Besides, we did not collect any means of social support, such as the number of friends, which would have provided much better operative variables for the intervention of PTSD. Moreover, our use of ORs may have overestimated the association of associated factors with mental health outcomes among nurses.

## CONCLUSIONS

We found that PTSD remained prevalent among Chinese nurses under the normalisation of COVID-19 pandemic prevention and control measures, with an incidence rate of 26.4%. Resilience and social support were factors associated with PTSD, while being married acted as a protective factor. Chinese hospital management must take measures to enhance the resilience and social support of nurses to reduce their symptoms of PTSD.
